# Detection of latent tuberculosis infection in healthcare workers

**DOI:** 10.3389/fmed.2026.1835510

**Published:** 2026-07-29

**Authors:** Anna Starshinova, Adilia Sabirova, Raul Sharipov, Ravil Tukfatullin, Aleksandr Panteleev, Michail Nazarenko, Dmitry Kudlay

**Affiliations:** 1 Almazov National Research Medical Center, St. Petersburg, Russia; 2 Laboratory of Probabilistic Methods in Analysis, Faculty of Mathematics and Computer Science, St. Petersburg State University, St. Petersburg, Russia; 3 Department of Medicine, Bashkir State Medical University of the Ministry of Health of the Russian Federation, Ufa, Russia; 4 Medical Department, St. Petersburg State Medical University Named After Academician I.P. Pavlov, St. Petersburg, Russia; 5 St. Petersburg Research Institute of Phthisiopulmonology, St. Petersburg, Russia; 6 St. Petersburg State Budgetary Healthcare Institution "City Tuberculosis Dispensary", St. Petersburg, Russia; 7 Department of Pharmacology, Institute of Pharmacy, I.M. Sechenov First Moscow State Medical University, (Sechenov University), Moscow, Russia; 8 FSBI SSC Institute of Immunology FMBA of Russia, Moscow, Russia; 9 Department of Pharmacognosy and Industrial Pharmacy, Faculty of Fundamental Medicine, Lomonosov Moscow State University, Moscow, Russia

**Keywords:** contact persons, Diaskintest®, immunodiagnostics, latent tuberculosis infection, medical staff, recombinant tuberculosis antigen test, risk groups

## Abstract

**Introduction:**

Identification of tuberculosis infection at different stages following exposure to a patient with tuberculosis is essential for improving epidemiological control, particularly during pathogen elimination and the stage of latent tuberculosis infection (LTBI). Detection of LTBI enables timely preventive interventions that may reduce the incidence of active tuberculosis. Healthcare workers represent an important risk group in whom LTBI screening using modern immunodiagnostic methods is required.

**Objective:**

To compare the prevalence of latent tuberculosis infection among healthcare workers in the general healthcare system, healthcare workers in tuberculosis facilities, and individuals with documented contact with patients with tuberculosis.

**Subjects and methods:**

A prospective comparative study was conducted including healthcare workers of the general healthcare system (*n* = 49), healthcare workers of tuberculosis institutions (*n* = 60), and individuals with documented contact with tuberculosis patients (*n* = 43). A control group consisted of healthy individuals without a history of contact with tuberculosis patients, clinical manifestations of tuberculosis, radiological signs of infection, or significant comorbidities (*n* = 51). Among healthcare workers in tuberculosis institutions, occupational contact with tuberculosis patients was reported in 91.7% (55/60; 95% CI 84.67–98.66). Among contact persons, exposure to a bacteriologically confirmed tuberculosis case occurred in 76.7% (33/43; 95% CI 64.12–89.37). All participants underwent immunodiagnostic testing using a test with recombinant tuberculosis antigen (ATR). Individuals with positive ATR results underwent comprehensive examination, including computed tomography of the chest, to exclude active tuberculosis. The prevalence of LTBI was defined as the proportion of positive ATR results. Statistical analysis was performed using χ^2^ tests with a significance threshold of *p* < 0.05 using Microsoft Excel 2019.

**Results:**

Positive ATR test results were detected in 8.2% of healthcare workers in the general healthcare system (95% CI 0.50–15.83), which did not significantly differ from the control group (5.8%; 95% CI 1.41–16.54; χ^2^ = 0.200, *p* = 0.655). In contrast, positive ATR results were significantly more frequent among healthcare workers in tuberculosis institutions (23.3%; 95% CI 12.63–34.04) compared with controls (5.8%; χ^2^ = 6.473, *p* = 0.011). The highest prevalence of positive ATR results was observed among contact persons (51.2%; 95% CI 36.22–66.10), which was significantly higher than in the control group (χ^2^ = 24.502, *p* = 0.001). Comparison between risk groups demonstrated that positive ATR results were significantly more common among healthcare workers in tuberculosis institutions than among healthcare workers in the general healthcare system (23.3% vs. 8.2%; χ^2^ = 4.502, *p* = 0.034). Furthermore, contact persons showed significantly higher positivity rates than both healthcare workers in the general healthcare system (51.2% vs. 8.2%; χ^2^ = 20.886, *p* = 0.001) and healthcare workers in tuberculosis institutions (51.2% vs. 23.3%; χ^2^ = 8.533, *p* = 0.004).

**Conclusion:**

The prevalence of LTBI detected using ATR immunodiagnostics was lowest among healthcare workers in the general healthcare system (8.2%), with values comparable to those observed in the control group (5.8%). Healthcare workers in tuberculosis institutions demonstrated a significantly higher prevalence of LTBI (23.3%), reflecting increased occupational exposure. The highest prevalence was observed among contact persons (51.2%), highlighting the substantial risk associated with close exposure to tuberculosis patients and emphasizing the importance of immunodiagnostic screening for early detection of LTBI.

## Introduction

Since 2015, the Russian Federation has demonstrated a sustained decline in the incidence of tuberculosis. In 2022, the incidence rate was 31.1 per 100,000 population; in 2023, 29.6; and in 2024, 26.9 per 100,000 population (1, 2). The continuing favorable epidemiological trend reflects the implementation of comprehensive organizational, preventive and diagnostic measures within the framework of the national tuberculosis control strategy. Nevertheless, further reductions in incidence are unlikely without optimization of approaches to the detection and management of latent forms of infection.

The identification of tuberculosis infection at its heterogeneous stages following primary exposure to an infectious case—ranging from pathogen clearance to the stage of latent tuberculosis infection (LTBI)—is of critical importance for improving the epidemiological situation. Timely diagnosis of LTBI enables the implementation of preventive interventions aimed at reducing the risk of progression to active disease and, consequently, decreasing the overall burden of tuberculosis (3–5).

Latent tuberculosis infection (LTBI) is defined as a state of persistent immune response to antigens of *Mycobacterium tuberculosis* in the absence of clinical, radiological or bacteriological evidence of active tuberculosis (6). According to several studies, the lifetime risk of developing active tuberculosis among infected individuals is estimated at 5–10%, with the highest risk occurring within the first 5 years following primary infection (7). This risk increases substantially in the presence of immunodeficiency, chronic comorbidities, or prolonged and intense exposure to an infectious source.

Currently, tuberculosis infection may be detected using immunological skin tests (the tuberculin skin test and the recombinant tuberculosis allergen skin test (Diaskintest®, Russia)) as well as laboratory-based interferon-gamma release assays (IGRAs). The overall sensitivity of these tests is broadly comparable, at approximately 80%, while specificity reaches around 95%. However, the specificity of the Mantoux test with 2 TU is considerably lower (approximately 60–65%), largely due to prior BCG vaccination and potential cross-reactivity with non-tuberculous mycobacteria (8). The use of more specific immunodiagnostic methods improves the accuracy of LTBI detection, particularly in high-risk groups.

Risk groups for tuberculosis were first systematically defined by the World Health Organization in the Guidelines on the Management of Latent Tuberculosis Infection (2014) and subsequently updated in Latent Tuberculosis Infection: Updated and Consolidated Guidelines for Programmatic Management (2018) (6). These documents identify individuals at increased risk of infection and progression to active disease, including healthcare workers. This professional group is exposed to an elevated occupational risk due to regular contact with patients, including those with undiagnosed tuberculosis, thereby justifying periodic LTBI screening using contemporary immunodiagnostic methods.

Data from international and domestic studies indicate considerable variability in the prevalence of LTBI among healthcare workers. For example, the prevalence of LTBI among staff of specialized tuberculosis facilities in Italy, assessed using the QuantiFERON-TB Gold In-Tube assay (QFT-GIT), was reported at 25% (9). Among healthcare workers in general medical services in the United Kingdom, the prevalence was 8% according to QFT-GIT results (10). In the Russian Federation, LTBI was identified in 32.7% of employees of forensic medical examination bureaux using the recombinant tuberculosis allergen skin test (11). These findings demonstrate substantial heterogeneity, likely attributable to differences in epidemiological context, occupational exposure and diagnostic strategies, which limits direct comparison across studies.

At the same time, close contacts of patients with tuberculosis are considered a particularly vulnerable group with respect to infection and the development of LTBI. In a systematic review by Fox G. J. et al., the pooled prevalence of LTBI among contacts ranged from 28.1 to 51.5%, depending on national income level, indicating a markedly higher burden compared with that observed among healthcare workers (12). Accordingly, systematic contact investigation in tuberculosis settings is essential for the timely identification of LTBI and the implementation of preventive measures (13).

The aim of the present study was to compare the prevalence of latent tuberculosis infection among healthcare workers in general medical services and close contacts of patients with tuberculosis.

## Materials and methods

A prospective comparative study was conducted in the Russian Federation between November 2025 and April 2026. Participants were consecutively recruited from healthcare institutions and tuberculosis services. The study included healthcare workers in general medical services (*n* = 49), healthcare workers employed in specialized tuberculosis facilities (*n* = 60), close contacts of patients with tuberculosis (*n* = 43), and healthy controls (*n* = 51). The study was performed at the V. A. Almazov National Medical Research Centre (Saint Petersburg, Russian Federation) and collaborating tuberculosis healthcare institutions.

According to the National Immunisation Programme of the Russian Federation, BCG vaccination is routinely administered during the neonatal period. Therefore, the majority of participants were presumed to have received BCG vaccination during childhood. Individual vaccination status was collected when available from medical documentation.

**Figure 1:**
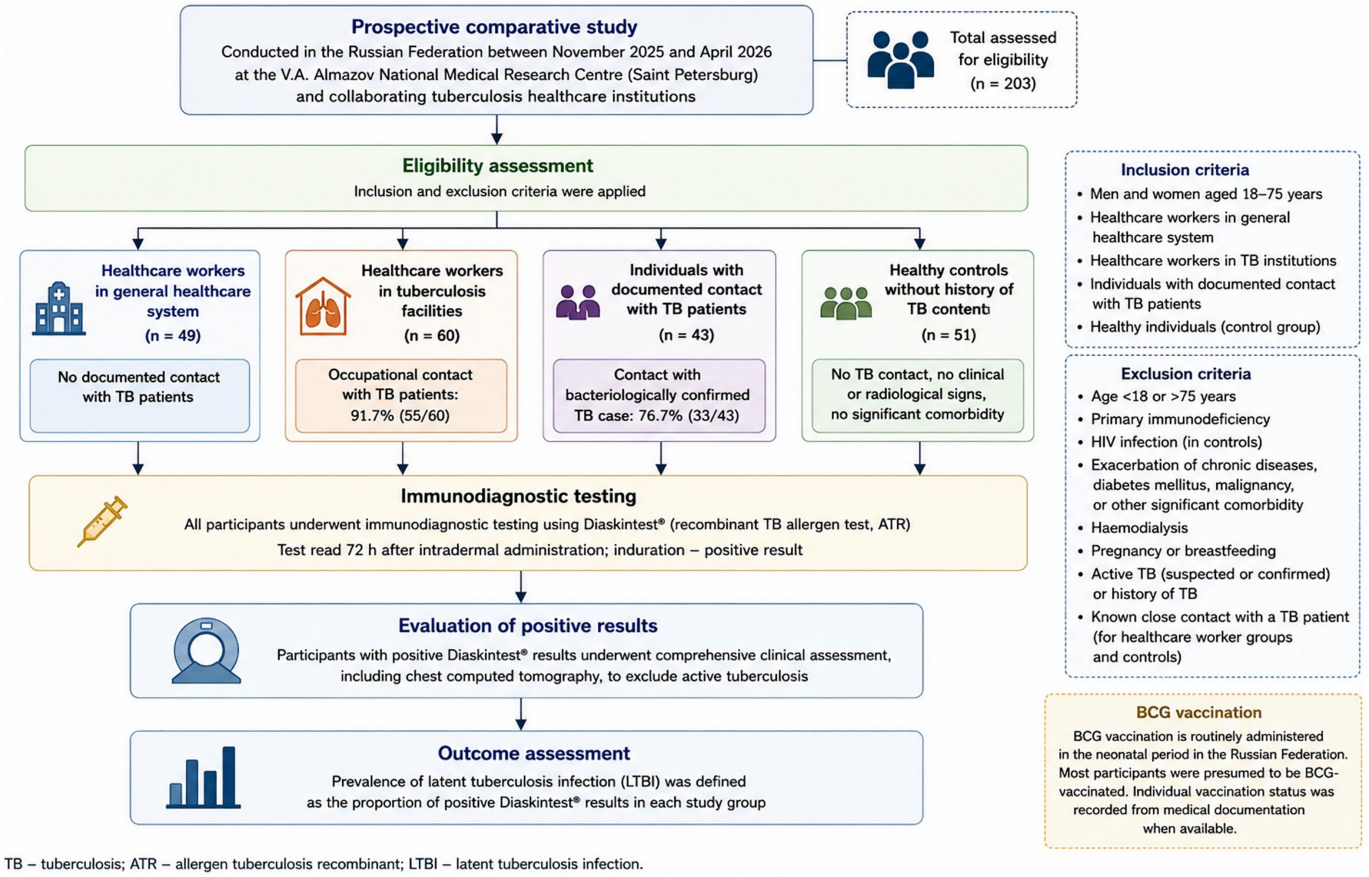
Study design.

The control group (*n* = 51) consisted of healthy individuals without contact with a patient with tuberculosis, without clinical or radiological signs of tuberculosis infection, and without concomitant pathology. The inclusion of a control group was necessary to identify statistically significant differences between immunodiagnostic results in the comparison groups and those obtained in healthy individuals. Allocation of participants to study groups was performed in accordance with the study design (Figure 1).

### Inclusion criteria for the comparison groups

1 Age between 18 and 75 years inclusive;
2 Healthcare workers in general medical services without a documented history of contact with a patient with tuberculosis;
3 Healthcare workers employed in specialized tuberculosis facilities;
4 Individuals with a documented history of household contact with a patient with tuberculosis;
5 Individuals with a documented history of occupational contact with a patient with tuberculosis.

### Exclusion criteria

1 Age below 18 years or above 75 years;
2 Individuals with immunosuppression, including:

- Primary immunodeficiency;
- People living with HIV;
- Receipt of immunosuppressive therapy for any indication (e.g., autoimmune diseases, post-organ or tissue transplantation, etc.);
- Patients undergoing hemodialysis;

3 Individuals with malignant neoplasms;
4 Individuals with suspected active tuberculosis, confirmed tuberculosis, or a previous history of tuberculosis.

### Study population

In all study groups, females predominated (Table 1). The mean age in the general medical healthcare worker group was 43 years (95% CI [38.07; 47.93]); in the specialized tuberculosis healthcare worker group, 40.9 years (95% CI [37.00; 44.80]); in the contact group, 41.7 years (95% CI [36.41; 49.99]); and in the healthy control group, 39.3 years (95% CI [36.81; 41.79]).

**Table 1:** Distribution of subjects by sex and age.

Comparison group	Men (%)	95% CI	Women (%)	95% CI	Mean age (years)	95% CI
Healthcare workers in general medical services (*n* = 49)	16.3	[5.98; 26.68]	83.7	[73.32; 94.02]	43.0	[38.07; 47.93]
Healthcare workers in specialized tuberculosis facilities (*n* = 60)	26.7	[15.48; 37.86]	73.3	[62.14; 84.52]	40.9	[37.00; 44.80]
Close contacts (*n* = 43)	44.2	[29.34; 59.03]	55.8	[40.97; 70.66]	41.7	[36.41; 49.99]
Healthy controls (*n* = 51)	39.2	[25.82; 52.62]	60.8	[47.38; 74.18]	39.3	[36.81; 41.79]

All healthcare workers in general medical services (*n* = 49) included in the study had no documented contact with a patient with active tuberculosis. Among healthcare workers in specialized tuberculosis facilities, 91.7% (55/60; 95% CI [84.67; 98.66]) had documented occupational contact with a patient with tuberculosis. All individuals in the control group (*n* = 51) had no documented contact with a patient with tuberculosis.

Among close contacts, 62.8% (27/43; 95% CI [48.34; 77.24]) had household contact with a family member; 34.9% (15/43; 95% CI [20.64; 49.13]) had occupational contact; and in 2.3% (1/43; 95% CI [0.06; 12.29]) the type of contact was not specified in the medical documentation. In 76.7% of cases (33/43; 95% CI [64.12; 89.37]), close contacts had exposure to a bacteriologically confirmed case.

### Diagnostic methods

All participants underwent detailed clinical history-taking and assessment of complaints. A standard diagnostic work-up was performed in all individuals, including chest radiography and the recombinant tuberculosis allergen skin test.

Diaskintest® (Generium, Russian Federation) is an intradermal diagnostic test containing recombinant *Mycobacterium tuberculosis*-specific antigens ESAT-6 and CFP-10. These antigens are encoded within the RD1 genomic region and are absent from *Mycobacterium bovis BCG* vaccine strains and most environmental non-tuberculous mycobacteria.

The test was administered intradermally according to the manufacturer’s instructions. Results were evaluated after 72 h. The presence of induration was considered a positive result. Due to the use of ESAT-6 and CFP-10 antigens, Diaskintest demonstrates high specificity in BCG-vaccinated populations while maintaining good sensitivity for detecting tuberculosis infection.

### Statistical analysis

Confidence intervals for continuous variables (age) were calculated after determination of descriptive statistics (mean and standard deviation) using the one-sample Student’s *t*-test. For binomial variables, 95% confidence intervals were calculated using the Wilson score method, which provides more robust interval estimation in relatively small samples and in groups with low event frequencies. The confidence level was set at 95%. Graphical representation of confidence intervals was performed using the Box Plot function in Microsoft Excel 2019.

The prevalence of LTBI in the comparison groups was defined as the proportion of positive Diaskintest results expressed as a percentage, with statistical significance set at *p* < 0.05. Statistical significance between comparison groups and the control group was assessed using Pearson’s χ^2^ test. Differences were considered statistically significant at *p* < 0.05.

Statistical analysis and graphical data presentation were performed using Microsoft Excel 2019, Statistica 8.0 (StatSoft, USA), and GraphPad Prism 4.00 for Windows (GraphPad Prism Software Inc., USA).

## Results

Positive results of Diaskintest (Table 2) were observed more frequently among healthcare workers in general medical services than among healthy controls (8.2% (4/49) vs. 5.8% (3/51)); however, the difference was not statistically significant (Table 3; χ^2^ = 0.200, *p* = 0.655). This indicates that the prevalence of LTBI among healthy individuals and healthcare workers in general medical services was comparable.

**Table 2:** Results of immunodiagnostics using the recombinant tuberculosis allergen test (Diaskintest).

Comparison group	Positive results (*n*)	%	95% CI	Negative results (*n*)	%	95% CI
Healthcare workers in general medical services (*n* = 49)	4	8.2	[0.50; 15.83]	45	91.8	[84.17; 99.50]
Healthcare workers in specialized tuberculosis facilities (*n* = 60)	14	23.3	[12.63; 34.04]	46	76.7	[65.96; 87.37]
Close contacts (*n* = 43)	22	51.2	[36.22; 66.10]	21	48.8	[33.90; 63.78]
Healthy controls (*n* = 51)	3	5.8	[1.41; 16.54]	48	94.2	[83.46; 98.59]

**Table 3:** Statistical significance of differences in immunodiagnostic results between groups.

Comparison groups	χ^2^	*p*-value
General medical healthcare workers vs. healthy controls	0.200	0.655*
Specialized TB healthcare workers vs. healthy controls	6.473	0.011
Specialized TB healthcare workers vs. general medical healthcare workers	4.502	0.034
Close contacts vs. healthy controls	24.502	0.001
Close contacts vs. general medical healthcare workers	20.886	0.001
Close contacts vs. specialized TB healthcare workers	8.533	0.004

*Here and throughout this table, the critical χ^2^ value was 3.841.

Among healthcare workers in specialized tuberculosis facilities, positive Diaskintest results were identified significantly more frequently than among healthy controls (23.3% (14/60) vs. 5.8% (3/51), χ^2^ = 6.473, *p* = 0.011) and significantly more frequently than among healthcare workers in general medical services (23.3% (14/60) vs. 8.2% (4/49), χ^2^ = 4.502, *p* = 0.034).

Positive test results were detected significantly more often among close contacts compared with healthy controls (51.2% (22/43) vs. 5.8% (3/51), χ^2^ = 24.502, *p* = 0.001), compared with healthcare workers in general medical services (51.2% (22/43) vs. 8.2% (4/49), χ^2^ = 20.886, *p* = 0.001), and compared with healthcare workers in specialized tuberculosis facilities (51.2% (22/43) vs. 23.3% (14/60), χ^2^ = 8.533, *p* = 0.004).

**Figure 2:**
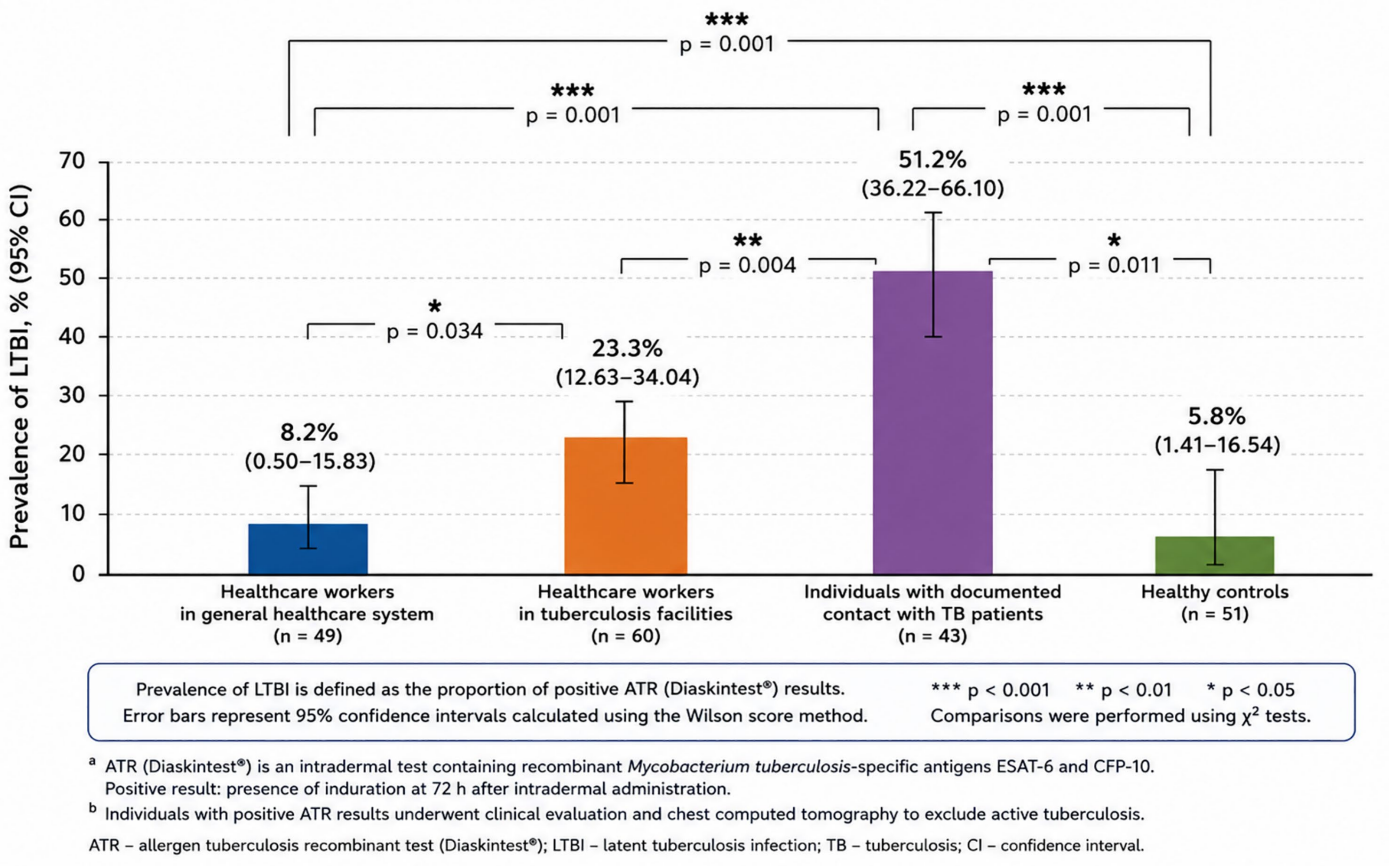
Prevalence of latent tuberculosis infection in the studied individuals, %.

Thus, early manifestations of tuberculosis infection, as evidenced by positive Diaskintest results, were observed in healthcare workers in general medical services (8.2%) no more frequently than in healthy individuals (5.8%), but significantly less frequently than in healthcare workers in specialized tuberculosis facilities (23.3%) and close contacts (51.2%) (Figure 2).

Furthermore, the prevalence of LTBI among healthcare workers in specialized tuberculosis facilities (23.3%), 91.7% (55/60) of whom had occupational contact with patients with tuberculosis, was substantially lower than among close contacts (51.2%), 76.7% (33/43) of whom had exposure to bacteriologically confirmed cases.

## Discussion

In the present study, the prevalence of LTBI among healthcare workers in general medical services was 8.2%, which is comparable to the 7.8% reported in a United Kingdom study using the QuantiFERON-TB Gold In-Tube assay (QFT-GIT) (10). A similar level of LTBI among healthcare workers in general medical settings was reported by Casas I. et al., who identified an LTBI prevalence of 31.4% based on the Mantoux test with 2 TU and 12.5% based on IGRA testing (14). The higher prevalence detected using the Mantoux test was attributed to prior BCG vaccination, which is known to reduce the specificity of tuberculin-based diagnostics. In Saudi Arabia, the prevalence of LTBI among healthcare workers based on immunodiagnostic testing was 10.8% (15), further supporting the notion that in non-specialized healthcare settings LTBI prevalence generally remains within a relatively moderate range when assessed using more specific assays.

At the same time, the prevalence identified in our study (8.2%) was higher than that reported by Bogorodskaya E. M. et al. in a 2018–2022 study assessing LTBI among employees of medical organizations under the Moscow Department of Health, where the mean prevalence over five years was 3.3% (16). This discrepancy may reflect regional epidemiological differences, variability in infection control practices, or differences in study design, including screening strategies and inclusion criteria.

Nevertheless, the 8.2% prevalence observed in our study remains substantially lower than that reported among forensic medical bureau employees in the Tyumen region (32.7%) (11) and among staff of specialized tuberculosis facilities in Italy (25% according to QFT-GIT) (9). These findings highlight the critical role of occupational exposure intensity in shaping LTBI epidemiology. Workers in phthisiology institutions and forensic medical settings are more likely to encounter undiagnosed or advanced cases of tuberculosis, often with high bacillary load, which increases the probability of transmission.

The variability in LTBI prevalence across studies may therefore be explained not only by differences in diagnostic approaches but also by heterogeneity in exposure patterns, including duration, frequency, and environmental conditions such as ventilation and adherence to infection control measures. In this context, the relatively moderate prevalence observed in our cohort suggests that exposure to *Mycobacterium tuberculosis* in general medical settings is likely to be intermittent and less intense.

Interpretation of LTBI prevalence across studies requires consideration of both biological and methodological factors. Differences in infection risk may arise from variations in exposure intensity, duration of contact, bacillary burden of index cases, environmental conditions, and host susceptibility. Equally important are methodological differences related to the diagnostic platform employed. The Mantoux tuberculin skin test, interferon-gamma release assays (IGRAs), and recombinant tuberculosis allergen-based tests such as Diaskintest® differ substantially in antigen composition, sensitivity, specificity, and susceptibility to confounding by prior BCG vaccination.

The Mantoux test utilizes purified protein derivative (PPD), which contains antigens shared by *Mycobacterium tuberculosis*, BCG strains, and several environmental mycobacteria. Consequently, specificity may be reduced in BCG-vaccinated populations, potentially leading to overestimation of LTBI prevalence.

In contrast, both IGRAs and Diaskintest® employ ESAT-6 and CFP-10 antigens that are absent from BCG strains, resulting in substantially improved specificity. However, differences remain between these methods because IGRAs assess interferon-*γ* production *in vitro*, whereas Diaskintest® measures a delayed-type hypersensitivity reaction *in vivo*.

Therefore, direct comparison of LTBI prevalence estimates across studies using different diagnostic methods should be interpreted cautiously. Apparent epidemiological differences may partly reflect methodological variability rather than true differences in infection burden (16).

Importantly, all healthcare workers included in our study had no documented history of contact with patients with active tuberculosis. This supports the assumption that the detected LTBI cases may be attributable to unrecognized or incidental occupational exposure, as well as possible community-acquired infection. The latter factor should not be underestimated, particularly in regions with ongoing tuberculosis transmission, where healthcare workers may share similar exposure risks with the general population.

The 8.2% LTBI prevalence identified in our cohort suggests that contacts between healthcare workers in general medical services and patients with tuberculosis are likely to be sporadic or short-term. However, the presence of LTBI in this group indicates that even limited or indirect exposure may be sufficient for transmission under certain conditions, especially in cases of delayed diagnosis or inadequate infection control practices.

Given that none of the healthcare workers in general medical services had a documented history of contact with a tuberculosis patient, it would not be expected that their LTBI prevalence would approximate that of close contacts, 76.7% of whom had confirmed exposure to a bacteriologically positive index case. Nonetheless, healthcare workers in general medical services constitute a recognized occupational risk group for the development of active tuberculosis. The magnitude of the risk of progression from LTBI to active disease in this population remains insufficiently characterized, representing a significant gap in current knowledge and a concern for both occupational and public health (17).

From a pathophysiological perspective, the risk of progression from LTBI to active tuberculosis is influenced by multiple host-related factors, including immune status, comorbidities (e.g., diabetes mellitus), and age, as well as infection-related factors such as bacterial load and recency of infection. In healthcare workers, repeated low-dose exposures may lead to a pattern of immune sensitization distinct from that observed in close contacts, which could potentially affect both diagnostic test performance and disease progression dynamics.

A positive trend has been observed in several countries where tuberculosis prevention strategies targeting healthcare workers have been implemented. For example, in Brazil, a hospital-based study demonstrated a substantial reduction in tuberculosis incidence among healthcare workers following the introduction of infection control measures, from 100 per 100,000 in 2005–2011 to 26.2 per 100,000 in 2012–2018 (18). These findings provide indirect evidence that effective infection prevention strategies can significantly reduce both transmission and subsequent LTBI burden in healthcare settings.

The World Health Organization recommends using the ratio of tuberculosis incidence among healthcare workers to that in the general adult population as a key indicator of infection control effectiveness. Ideally, this ratio should approach 1 in settings with adequate preventive measures. However, the reporting of 17,449 tuberculosis cases among healthcare workers from 73 countries in 2023 (19) indicates that occupational transmission remains a substantial global problem, necessitating further optimization of both preventive and diagnostic strategies.

The LTBI prevalence among close contacts in our study (51.2%) is consistent with previously reported data, including the 42.5% reported by Chang V. et al. (20). The high prevalence in this group is likely driven by the intensity of exposure, particularly given that 76.7% of contacts in our cohort were exposed to bacteriologically confirmed index cases. This underscores the importance of bacillary burden as a key determinant of transmission risk.

Reichler et al. (21) further demonstrated that LTBI prevalence among contacts is influenced by a complex interplay of factors, including cumulative exposure time, characteristics of the index case (e.g., cavitary disease, smear positivity), and host-related variables. Notably, the observed increase in LTBI probability by approximately 8% for each additional 250 h of exposure highlights the quantitative relationship between exposure duration and infection risk. At lower levels of exposure (<250 h), the absence of a clear linear trend suggests the presence of threshold effects or variability in individual susceptibility.

Recent studies by Choi Y. et al. and Godoy S. et al. (22, 23) have reinforced the critical role of prolonged exposure in determining LTBI risk. In our study, 62.8% of contacts had household exposure, which typically involves repeated and prolonged interactions in enclosed environments. This likely contributed substantially to the high LTBI prevalence observed in this group and is consistent with established epidemiological patterns.

An important aspect of interpreting the obtained results concerns the selection and performance of immunodiagnostic methods for LTBI detection across different risk groups. Current international guidelines emphasize that test selection should be individualized based on epidemiological context, BCG vaccination status, and the pre-test probability of infection.

In BCG-vaccinated populations, IGRA-based assays and recombinant tuberculosis allergen skin tests (such as Diaskintest) offer superior specificity compared with the Mantoux test, as they utilize antigens absent from BCG strains and most non-tuberculous mycobacteria. This is particularly relevant in occupational settings where repeated testing is performed, as it reduces the likelihood of false-positive results and unnecessary preventive interventions.

However, the interpretation of serial IGRA testing remains challenging due to the phenomenon of conversion and reversion, especially in low-incidence settings. These fluctuations may reflect biological variability, assay-related factors, or transient immune responses rather than true infection or clearance. Consequently, the establishment of standardized definitions for significant test changes is essential for reliable longitudinal monitoring.

In close contacts of infectious tuberculosis cases, immunodiagnostic testing demonstrates higher sensitivity due to recent exposure and heightened immune activation. The recommended strategy of repeat testing 8–12 weeks after the last exposure allows for the detection of delayed immune conversion, thereby improving diagnostic accuracy.

For healthcare workers in specialized tuberculosis facilities, periodic screening remains a cornerstone of occupational health programs. The frequency of testing should be guided by risk stratification, taking into account local epidemiology, job category, and documented exposure incidents. In contrast, in general medical settings, a more targeted approach may be appropriate, focusing on high-risk departments and individuals with potential exposure.

From a preventive medicine perspective, the identification of LTBI has direct implications for clinical management. Preventive therapy has been shown to significantly reduce the risk of progression to active tuberculosis, particularly among recent contacts and immunocompromised individuals. In healthcare workers, decisions regarding preventive treatment should be individualized, considering factors such as age, comorbidities, risk of hepatotoxicity, and likelihood of recent infection.

In addition, organisational measures—such as early identification of patients with suspected tuberculosis, prompt isolation, use of personal protective equipment, and adequate ventilation—remain fundamental components of infection control (24, 25). The integration of these measures with effective screening programs is essential to reduce both occupational exposure and LTBI prevalence.

The Russian Federation, the investigation and clinical implementation of these tests commenced in 2012. Russian researchers may be regarded as pioneers in the development of *in vivo* diagnostic approaches for latent tuberculosis infection. Notably, a research group from the Research Institute of Molecular Medicine at the I. M. Sechenov Moscow Medical Academy, led by Academician M. A. Pal’tsev and Corresponding Member of the Russian Academy of Sciences V. I. Kiselev, were the first to develop a novel skin test based on a recombinant tuberculosis allergen (Diaskintest), with successful clinical evaluation reported as early as 2008. The assay incorporates the antigens ESAT-6 and CFP-10, which are absent from *Mycobacterium bovis* BCG strains, thereby conferring a high degree of specificity and enabling differentiation between BCG-induced hypersensitivity and true *Mycobacterium tuberculosis* infection (26, 27). This represents a significant methodological advantage over the traditional tuberculin skin test in BCG-vaccinated populations.

Clinical studies have demonstrated that the specificity of Diaskintest lies within a confidence interval of 90–100%, with no evidence of cross-reactivity attributable to prior BCG vaccination and no documented non-specific allergic responses. Furthermore, administration of the test at a dose of 0.2 μg in 0.1 mL elicits a delayed-type hypersensitivity reaction in 98–100% of individuals with active tuberculosis or confirmed infection with *M. tuberculosis* (28, 29).

However, despite these favorable performance characteristics, several considerations warrant attention. As with other immunological assays, the diagnostic accuracy of Diaskintest may be influenced by host immune status, potentially leading to false-negative results in immunocompromised individuals. In addition, while high specificity supports its use in BCG-vaccinated populations, the sensitivity of the test in detecting early or low-burden infection requires further evaluation in diverse epidemiological settings. These factors should be taken into account when integrating Diaskintest into screening algorithms, particularly in populations with varying risk profiles and background tuberculosis incidence.

### Implications and future directions

Thus, optimization of LTBI diagnostics and control strategies in risk groups should include:

- the use of highly specific immunodiagnostic methods in BCG-vaccinated populations;
- targeted and periodic screening of occupational risk groups based on exposure assessment;
- systematic and timely contact investigation with repeat testing when indicated;
- development of standardized algorithms for interpreting serial test results;
- integration of immunodiagnostic findings into evidence-based decision-making for preventive therapy.

Future research should focus on longitudinal assessment of LTBI dynamics among healthcare workers, including rates of conversion, reversion, and progression to active disease. In addition, studies evaluating the cost-effectiveness of different screening strategies and preventive interventions in various epidemiological settings are warranted.

Overall, these approaches may enhance early detection of tuberculosis infection, reduce the risk of disease progression, and contribute to improved tuberculosis control both in healthcare settings and in the broader community.

### Limitations of the study

A number of limitations inherent to this study should be acknowledged. Firstly, the sample size was relatively modest, particularly within certain comparison groups. Secondly, a cross-sectional design was employed without longitudinal follow-up; consequently, progression from latent tuberculosis infection (LTBI) to active disease could not be evaluated.

Furthermore, multivariable logistic regression analysis was not performed due to the limited sample size and the comparatively small number of positive outcomes. Exposure intensity was characterized solely by documented contact history and could not be quantified in detail.

Finally, the research was conducted at a limited number of centers across the Russian Federation, which may restrict the generalizability of the findings.

## Conclusion

The present findings indicate that the lowest prevalence of LTBI, as determined by Diaskintest, was observed among healthcare workers in general medical services (8.2%), a level comparable to that of the control group (5.8%). The prevalence among healthcare workers in specialized tuberculosis facilities (23.3%) was significantly higher than among healthcare workers in general medical services, as confirmed by statistically significant differences. The highest LTBI prevalence was identified among close contacts (51.2%), significantly exceeding that in other risk groups, reflecting early manifestations of tuberculosis infection and underscoring the importance of immunodiagnostic methods for LTBI detection.

## Data Availability

The original contributions presented in the study are included in the article/supplementary material, further inquiries can be directed to the corresponding author.
